# Hairy cell leukemia: a specific 17-gene expression signature points to new targets for therapy

**DOI:** 10.1007/s00432-022-04010-4

**Published:** 2022-04-27

**Authors:** Elsa Maitre, Edouard Cornet, Agathe Debliquis, Bernard Drenou, François Gravey, Didier Chollet, Stephane Cheze, Mylène Docquier, Xavier Troussard, Thomas Matthes

**Affiliations:** 1 Normandie University, UNIROUEN, UNICAEN, INSERM1245, MICAH, Avenue de la côte de Nacre, 14033 Caen, France; 2 Laboratory Hematology, University Hospital Caen, Avenue de la Côte de Nacre, 14033 Caen cedex, France; 3 Department of Haematology, Groupe Hospitalier de la Région Mulhouse Sud Alsace, 20 avenue du docteur René laennec, 68100 Mulhouse, France; 4 Normandie University, UNIROUEN, UNICAEN, GRAM2.0, Avenue de la côte de Nacre, 14033 Caen, France; 5 iGE3 Genomics Platform, University Medical Center, Geneva University, 1211 Geneva, Switzerland; 6 Hematology Institute, University Hospital Caen, Avenue de la Côte de Nacre, 14033 Caen, France; 7 Hematology Service, Department of Oncology and Clinical Pathology Service, Department of Diagnostics, University Hospital Geneva, 1211 Geneva, Switzerland; 8 Department of Genetics and Evolution, Sciences III, Geneva University, 1205 Geneva, Switzerland

**Keywords:** Hairy cell leukemia, NF-KB pathway, Gene expression, RGS13, EBI3

## Abstract

**Background:**

Hairy cell leukemia (HCL) is a rare chronic B cell malignancy, characterized by infiltration of bone marrow, blood and spleen by typical “hairy cells” that bear the BRAFV600E mutation. However, in addition to the intrinsic activation of the MAP kinase pathway as a consequence of the BRAFV600E mutation, the potential participation of other signaling pathways to the pathophysiology of the disease remains unclear as the precise origin of the malignant hairy B cells.

**Materials and methods:**

Using mRNA gene expression profiling based on the Nanostring technology and the analysis of 290 genes with crucial roles in B cell lymphomas, we defined a 17 gene expression signature specific for HCL.

**Results:**

Separate analysis of samples from classical and variant forms of hairy cell leukemia showed almost similar mRNA expression profiles apart from overexpression in vHCL of the immune checkpoints *CD274* and *PDCD1LG2* and underexpression of *FAS*. Our results point to a post-germinal memory B cell origin and in some samples to the activation of the non-canonical NF-κB pathway.

**Conclusions:**

This study provides a better understanding of the pathogenesis of HCL and describes new and potential targets for treatment approaches and guidance for studies in the molecular mechanisms of HCL.

**Supplementary Information:**

The online version contains supplementary material available at 10.1007/s00432-022-04010-4.

## Introduction

Hairy cell leukemia (HCL) is a rare mature B cell malignancy, with an incidence of approximately 0.3 per 100,000 and is characterized by an accumulation of malignant B-lymphoid cells with a particular “hairy” appearance. The classical HCL (cHCL) is characterized by the expression of CD19, CD20, CD22, FMC7 and also CD11c, CD25, CD103, CD123 expression on the surface of the hairy cells (Swerdlow et al. 2017). In the variant HCL (vHCL), the cells are typically CD25neg (Swerdlow et al. 2017). In 2011, E. Tiacci et al*.* showed that 100% of cHCL presented the oncogenic *BRAF* mutation V600E and proposed that the mutation was a driver mutation (Tiacci et al. 2011). Interestingly, none of the vHCL samples sequenced so far had shown the BRAF mutation (Waterfall et al. 2013). In our mRNA study using NanoString technology on a code Set of 290 genes, we demonstrate a specific mRNA signature of cHCL and vHCL based on 17 genes. We find minor differences between the classical and the variant form, confirm the specific expression of several already published genes such as *CCND1* and *ITGAE*/CD103, and describe new ones, suggesting an activation of the non-canonical NF-kB pathway in some samples, and a post-germinal memory B cell origin of hairy cell leukemia cells.

## Methods

### Patients

Samples were obtained from 13 newly diagnosed HCL patients (11 cHCL, 2 vHCL) of the Hematology Services of the Geneva, Mulhouse and Caen hospitals with > 30% of abnormal hairy cells in the peripheral blood. All participants were recruited following written informed consent and after approval of the research protocol by the local ethics committees. All patient data were analyzed anonymously. cHCL and vHCL were diagnosed in accordance with the WHO 2016 classification based on clinical criteria, cytology, immunophenotyping and presence of *BRAF*^*V600*^ (Swerdlow et al. 2017). All the relevant patient information are presented in Online Resource Table S1.

From each sample, mononuclear cells were obtained by FICOLL gradient centrifugation and then (a) either lysed directly in RNA lysis buffer (Qiagen, Venlo, Netherlands) and stored at − 80 °C (Geneva), or (b) resuspended in DMSO, stored in liquid nitrogen, thawed for the present study, and then put into RNA lysis buffer (Mulhouse), or (c) lysed in RNA lysis buffer, followed by RNA extraction and storage at − 80 °C (Caen).

Normal blood samples were obtained from healthy blood donors of the Geneva blood transfusion center. Mononuclear cells (nMNC) were prepared by FICOLL gradient centrifugation and contained between 12 and 30% of normal mature B cells. CD19 + B cells were further enriched using a CD19pos Selection Kit from Stemcell Technologies, according to the manufacturer’s instructions. Purity of the isolated B cell populations was verified by flow cytometry with specific anti-CD19 and anti-CD20 antibodies and was > 95% in all cases (nB; not shown).

### mRNA analysis

We performed an extensive literature search and extracted a set of 290 B cell lymphoma-specific genes from published articles and public databases with the aim of defining a set of genes described to be either over- or underexpressed in HCL compared to other mature B cells neoplasms or compared to normal B lymphocytes (Basso et al. 2004; Cornet et al. 2015). Nine normalization genes were added to this list to obtain a set of 299 genes which was used for the analysis with the nCounter system (Online Resource Table S2). mRNA analysis and counting were performed with NanoString technologies, according to the manufacturer’s protocol (Nanostring H Technologies, Seattle, WA, USA). Each mRNA species was extracted, pre-processed, analyzed with the nCounter system then normalized using R (NanostringNorm R package; version 1.2.0, http://cran.r-project.org/package=NanoStringNorm) (Waggott et al. 2012).

Raw data were adjusted against the geometric mean of spiked-in positive probes to account for differences in hybridization and recovery, background-corrected (background = mean of negative probes + 2 SD), and normalized against housekeeping genes (*ACTB*, *TBP*, *RPL19*, *RPLP0*, *G6PD*, *ABCF1*, *B2M*, *TPT1*, *RPS23*) to account for differences in sample content. The technical specificities of the NanoString technology (linearity, reproducibility, sensitivity, etc.) have all been previously described (Geiss et al. 2008; Beaume et al. 2011; Fernandez et al. 2012).

The data were submitted to GEO and can be accessed via Access Number GSE161279.

Since our samples were constituted of total white blood cells containing malignant B cells in various percentages together with contaminating other white blood cells such as monocytes and T lymphocytes, we used a special deconvolution algorithm to extract B cell-specific signatures from the total mRNA signatures obtained. We compared the mean of the total counts obtained from the samples of each group of samples (HCL, nMNC and purified nB cells) and considered only those genes that fulfilled the arbitrary criteria of an expression level  ≥  20 counts, and a  ≥  twofold change difference in expression as preferentially expressed by one group or not. In this way, the gene set preferentially expressed by normal B cells (nB) was obtained by the comparison of nB versus nMNC; the gene set overexpressed in HCL by the comparison of HCL with nB and nMNC samples, and the gene set underexpressed by HCL by the comparison of HCL samples with nMNC samples.

Gene Ontology enrichment was performed with the open source Enrichr analysis tool (http://amp.pharm.mssm.edu/Enrichr) (Chen et al. 2013). Gene Set Enrichment Analysis (GSEA) was conducted using GSEA software (Subramanian et al. 2007).

### Statistical analysis

R version 3.4.2 software was used for statistical calculations and data presentation. For gene expression analyses, genes with expression levels ≥ 20 counts, fold-changes > or < 2 compared to reference values, with corrected *p* values < 0.05 (according to Benjamini correction for multiple testing (Benjamini and Hochberg 1995)) were considered as significant.

## Results

Samples from hairy cell leukemia patients were analyzed with the aim to develop molecular mRNA signatures specific for this lymphoma and to gain new insights into the pathophysiology of the disease.

### Patient characteristics

Eleven samples were obtained from patients with the cHCL defined by cytology, immunophenotyping and presence of the BRAF^V600E^ mutation. Two vHCL were studied and did not show the *BRAF*^V600E^ mutation, but an activating *MAP2K1* mutation (Online Resource Table S1).

### mRNA-specific signature in hairy cell leukemia

**Fig. 1:**
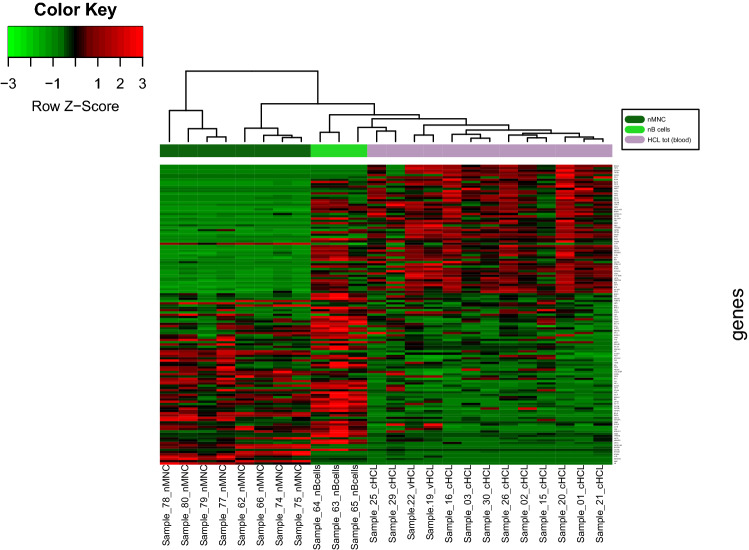
Unsupervised clustering analysis. Thirteen samples from peripheral blood samples of HCL patients were analyzed in parallel to 8 samples from nMNC (normal mononuclear cells) and 3 samples of purified nB cells (normal B cells). The heatmap shows the expression of 290 genes expressed in the 13 HCL samples compared to nMNC and nB cell samples

**Fig. 2:**
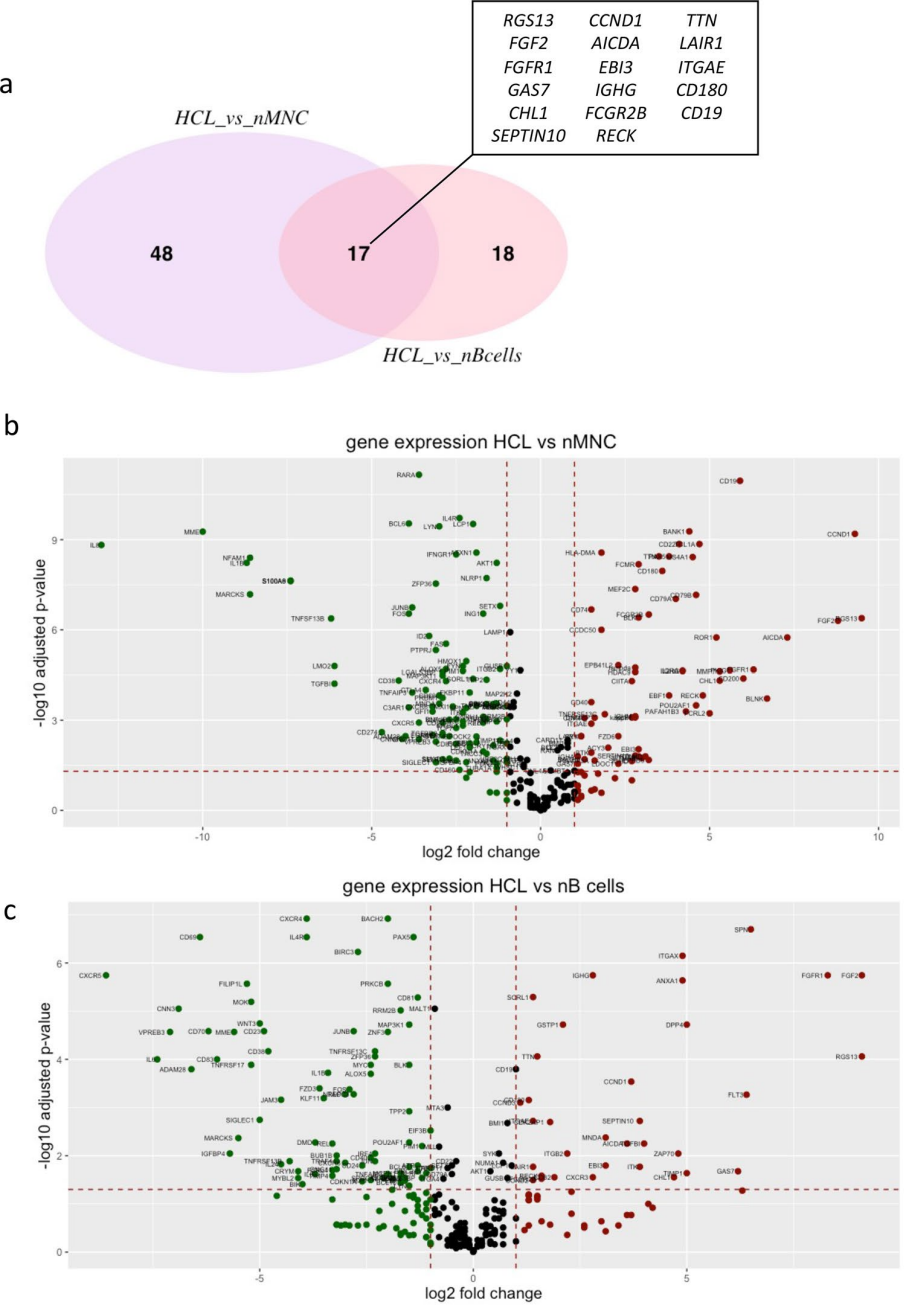
Venn diagram and volcano plots of differentially expressed genes. **a** The Venn diagram shows the number of genes overexpressed in HCL samples, compared to nMNC and nB cell samples, respectively. **b** Volcano plot of genes expressed differentially in 13 HCL samples compared to 8 nMNC samples. **c** Volcano plot of genes expressed differentially in 13 HCL samples compared to 3 nB cell samples. To be considered preferentially expressed, genes had to fulfill the following criteria: expression levels ≥ 20 counts, a ≥ twofold difference in expression between the sample groups, and a corrected *p* value ≤ 0.05

We studied the expression of 290 genes described to be crucial in the pathophysiology of B cell lymphomas based on information of published articles and public databases. Thirteen samples from HCL patients, eight samples from normal blood donors (nMNC) and three samples of sorted normal B cells (nB) were analyzed with the Nanostring technology. Quantitative, relative values for mRNA copy numbers for all 290 genes in all the samples tested were obtained (GEO Access No. GSE161279). As the patient samples did not consist of sorted B cells but of total nucleated white blood cells, we developed a special deconvolution algorithm which allowed us to obtain B cell-specific mRNA signatures despite the presence of contaminating other cells in the samples (see “Methods”). Using this algorithm, we compared the expression of genes in HCL samples to the expression in nMNC and in sorted nB. In the samples of sorted nB cells, typical B cells genes were found to be highly expressed, such as surface markers *CD19*, *CD22* and *CD79A/B*, immunoglobulin genes, and B cell transcription factors such as *PAX5*. Out of the total 290 genes analyzed 107 genes were thus considered to be B cell-specific (expression level  ≥  20 counts,  ≥  twofold increase in expression compared to nMNC samples, *p* value ≤ 0.05) (Online Resource Table S2). Unsupervised clustering resulted in a clear separation of the 13 HCL from the nMNC and nB (Fig. 1). In this unsupervised analysis, vHCL samples (samples 19 and 22) were intermixed with cHCL, suggesting a comparable gene expression signature. As shown in Online Resource Fig. S1, there was a close correlation in differentially expressed genes between cHCL and vHCL when comparing to nB (*r* = 0.82, *p* = 2.2 × 10^− 16^). To find among the 290 genes that could differentiate most effectively between HCL, nMNC and nB, we compared the mean of all the gene counts from the 13 HCL samples to the mean from the nMNC samples and to the mean of the nB, selecting only genes, that fulfilled the criteria mentioned above. Figure 2a shows the intersection for the genes overexpressed in HCL compared to nMNC (65 genes) and compared to nB cells (35 genes); Fig. 2b and c shows the graphical representation of gene expression by volcano plots, and the Online Resource Table S4a, the list of genes. Among the overexpressed genes, 17 were common to both lists (HCL vs nMNC and HCL vs nB) (Table 1): *CD19*, *IGHG*, *CCND1*, *ITGAE*, *GAS7*, *FGF2*, *FGFR1*, *RECK*, *TTN*, *CHL1*, *SEPINT10*, *RGS13*, *AICDA*, *EBI3*, *CD180*, *LAIR1*, and *FCGR2B.* As expected, this set of 17 genes contained genes well known to be expressed in HCL (*CCND1*, *ITGAE*/CD103) and served as internal quality control to validate the analysis (Matutes et al. 1994; Bosch et al. 1995).

**Table 1:** List of 17 genes overexpressed in HCL samples

Name	Description	Average fold change compared to nB	Adjusted *p* value compared to nB	Average fold change compared to nMNC	Adjusted *p* value compared to nMNC	Previously described in HCL		References
Cytokines/chemokines and their modulators								
EBI3	Epstein–Barr Virus Induced 3	3.1	1.60E-02	2.9	9.20E-03	**No**		
Differentiation								
RGS13	Regulator of G protein signaling 13	9.1	8.70E-05	9.5	4.10E-07	**No**		Vanhentenrijk et al. (2004) (CGH/CESH)
AICDA	Activation-induced cytidine deaminase	3.6	5.60E-03	7.3	1.80E-06	Yes	*	Navarro et al. (2017), Hockley et al. (2010)
BCR pathway/B cell activation								
CD19		1	1.60E-04	5.9	1.10E-11	Yes	*	Matutes et al. (1994)
Cell adhesion/migration/cytoskeleton								
FGF2	Fibroblast growth factor 2 = basic-FGF	9.1	1.80E-06	8.8	5.00E-07	Yes	*, #	Basso et al. (2004), Gruber et al. (1999)
FGFR1	Fibroblast growth factor receptor 1	8.3	1.80E-06	6.3	2.10E-05	Yes	#	Basso et al. (2004)
GAS7	Growth arrest specific 7	6.2	2.10E-02	1.1	2.80E-02	Yes	#	Basso et al. (2004)
CHL1	Cell adhesion molecule L1 like	4.7	2.80E-02	5.3	4.80E-05	**No**		
RECK	Reversion inducing cysteine-rich protein with Kazal motifs	1.6	2.60E-02	4.8	1.50E-04	Yes	#	Basso et al. (2004)
ITGAE	CD103	1.4	1.90E-03	1.5	1.30E-03	Yes	*	Matutes et al. (1994)
SEPTIN10	Septin family	3.9	1.90E-03	2.8	1.50E-02	**No**		
TTN	Titin	1.5	8.70E-05	3.5	3.60E-09	**No**		
Proliferation								
CCND1	Cyclin D1, cell cycle	3.7	2.90E-04	9.3	6.40E-10	Yes	*, #	Bosh et al. (1995), Basso et al. (2004)
Immunoreceptor/cell surface receptor/antigen presentation								
IGHG	Immunoglobulin heavy constant gamma	2.8	1.80E-06	4.2	2.30E-05	Yes	*	Golomb et al. (1982)
FCGR2B	Fc fragment of IgG receptor IIb, CD32	1.6	3.00E-02	3.2	3.10E-07	**No**		
LAIR1	Leukocyte-associated immunoglobulin-like receptor 1, CD305	1.4	1.70E-02	1.2	3.40E-03	Yes	*	Garnache Ottou et al. (2014)
CD180	Belongs to the family of Toll-like receptors	1.3	7.00E-04	3.6	1.10E-08	Yes	*	Favre et al. (2018)

Nanostring analysis showed mRNAs of 17 genes overexpressed in HCL samples compared to normal B cell (nB) and normal MNC samples (nMNC), fulfilling the following criteria: expression levels ≥ 20 counts, a ≥ twofold difference in expression between sample groups, and a corrected *p* value ≤ 0.05

Genes were grouped according to their function in different cellular activities

^#^Previously described in gene expression analysis

^*^Previously described in phenotypic analysis

Enrichment analysis with gene ontology (GO) annotation showed that among the 65 overexpressed genes in HCL compared to nMNC were included genes characteristically expressed in B cells, such as B cell activation genes (*CD79B*, *CD79A*, *CD40*, *MEF2C*, *BANK1*, *BLNK*, *BTK*, *MS4A1*, *HDAC9* and *AICDA*), B cell receptor pathway genes (*BLK*, *CD79B*, *IGHM*, *CD79A*, *MEF2C*, *SYK*, *CD19*, *IGHD* and *BTK*), genes with a negative regulatory role in cell cycle arrest (*CCND1*, *CDK4*, and *SETMAR*) and genes implicated in the regulation of the MAPK cascade (*EPHB6*, *CD74*, *CD40*, *SYK*, *ROR1* and *FGFR*) (Online Resources Fig. S2a and Table S4a). Among the 35 overexpressed gene comparing HCL to nB, enrichment analysis with GO showed in HCL enrichment of groups of genes mostly involved in positive regulation of protein serine/threonine kinase activity (CCND3, *CCND2*, *CCND1*, *FLT3*, *FGF2* and *FGFR1*) or in extracellular matrix organization (*ITGB2*, *ITGAX*/CD11c, *ITGAE*/CD103, *TIMP1*, *TGFBI*, *FGF2* and *RECK*) (Online Resource Fig. S2b). In addition, gene set enrichment analysis (GSEA) of genes involved in positive regulation of mitotic cell cycle and positive regulation of MAP kinase activity were significantly overexpressed in HCL samples compared to normal cells (Online Resource Fig. S2c).

Eighty genes were found to be specifically underexpressed in HCL compared to normal B cell samples (Fig. 2 and Online Resource Table S4b). The most underexpressed genes were *CXCR5*, *IL-6*, VPREB3, *CNN3*, and *ADAM28* and the surface markers *CD69*, *CD70* and *CD83*. According to the enrichment analysis with GO, groups of underexpressed genes were part of the cytokine-mediated signaling and the regulation of I-κB kinase/NF-κB signaling pathways, with underexpression of *CD40*, *PRKCB*, *IL1B*, *REL*, *TNFAIP3*, *BCL10*, *NLRP1*, *BIRC2* and *BIRC3* genes (Online Resources Fig. S2b and Table S4b).

### Genes differentially expressed in cHCL and vHCL

**Fig. 3:**
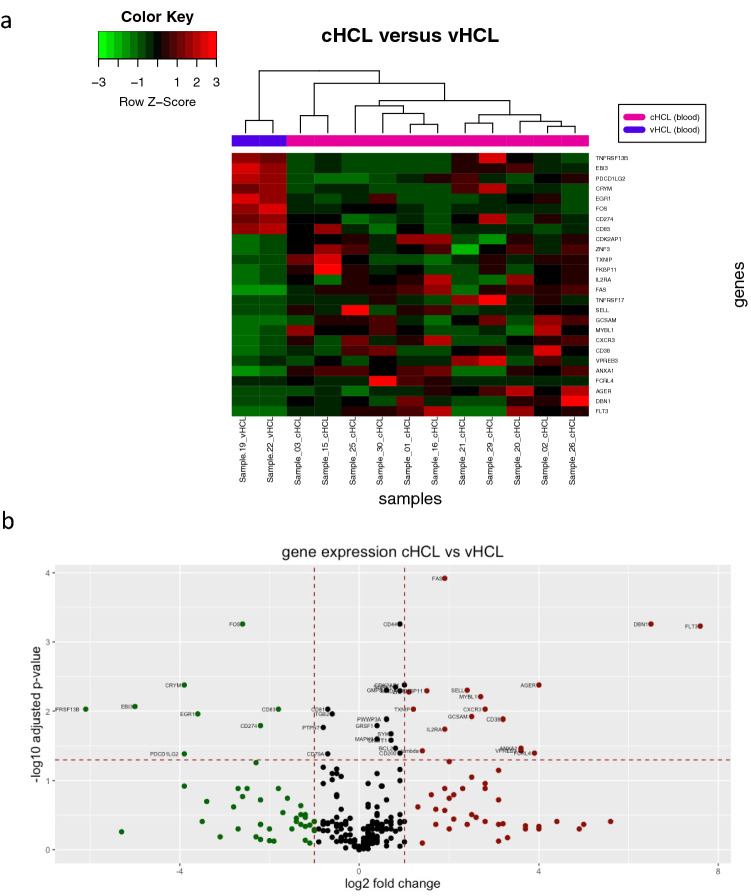
Comparison between cHCL and vHCL samples. **a** Unsupervised hierarchical clustering of gene expression profiles generated from cHCL (*n* = 11) and vHCL (*n* = 2) blood samples. **b** Volcano plot of genes expressed differentially in 11 cHCL samples compared to 2 vHCL blood samples. To be considered preferentially expressed, genes had to fulfill the following criteria: expression levels ≥ 20 counts, a ≥ twofold difference in expression, and a corrected *p* value ≤ 0.05

Using unsupervised clustering, we identified a similar signature between cHCL and vHCL (Fig. 1) with minor differences, when comparing the gene expression (Fig. 3 and Table 2). In addition to expected overexpressed genes in cHCL compared to vHCL (*ANXA1* and *IL2RA*), we also noted an overexpression of genes involved in chemotaxis/adhesion (*CXCR3*, *SELL*/CD62L, *FLT3*) and in apoptosis regulation (*FAS*, *CDK2AP1*). Among underexpressed genes in cHCL, we found immune checkpoint regulators, such as *CD274* (PD-L1) and *PDCD1LG2* (PD-L2) and the TNF receptor *TNFRSF13B*.

**Table 2:** List of genes expressed preferentially in cHCL compared to vHCL

Genes	Adj *p* value	Log2(FC) cHCL	Log2(FC) vHCL
Overexpressed in cHCL			
FLT3	0.00059	7.6	− 7.6
DBN1	0.00055	6.5	− 6.5
AGER	0.0042	4	− 4
FCRL4	0.04	3.9	− 3.9
ANXA1	0.034	3.6	− 3.6
VPREB3	0.037	3.6	− 3.6
CD38	0.013	3.2	− 3.2
CXCR3	0.0094	2.8	− 2.8
MYBL1	0.0062	2.7	− 2.7
GCSAM	0.012	2.5	− 2.5
SELL	0.005	2.4	− 2.4
FAS	0.00012	1.9	− 1.9
IL2RA	0.018	1.9	− 1.9
FKBP11	0.0051	1.5	− 1.5
TXNIP	0.0094	1.2	− 1.2
ZNF3	0.0053	1.1	− 1.1
CDK2AP1	0.0042	1	− 1
Overexpressed in vHCL			
CD83	0.0094	− 1.8	1.8
CD274	0.016	− 2.2	2.2
FOS	0.00055	− 2.6	2.6
EGR1	0.011	− 3.6	3.6
CRYM	0.0042	− 3.9	3.9
PDCD1LG2	0.041	− 3.9	3.9
EBI3	0.0086	− 5	5
TNFRSF13B	0.0094	− 6.1	6.1

List of genes expressed differentially in 11 cHCL blood samples compared to 2 vHCL blood samples. Genes either over- or underexpressed in cHCl compared to vHCL were listed if they fulfilled the following criteria: expression levels ≥ 20 counts, a ≥ twofold difference in expression, and a corrected *p* value ≤ 0.05

### A NF-κB non-canonical pathway dysregulation in HCL

The analysis of the list of genes underexpressed in HCL compared to normal B cells showed several genes belonging to the NF-κB pathway, such as *PRKCB*, *IL1B*, *REL*, *BCL10*, and *NLRP* of the canonical and BIRC2 and BIRC3 of the non-canonical pathway. To assess whether the non-canonical pathway was intrinsically activated, we studied the ratio of the p100 protein, the p52 precursor, and the mature p52 protein. When the pathway is activated, p100 is processed and the ratio falls below one, as in the cell line JVM-2, a mantle cell lymphoma cell line. Because of lack of material, only seven cHCL samples could be tested. 4/6 cHCL and 1/1 vHCL expressed a p100/p52 ratio < 1, in line with p100 processing and activation of the non-canonical NF-κB pathway (Online Resource Fig. S3).

## Discussion

Gene expression studies have extensively been performed in hematologic neoplasms and B cell lymphomas but few studies were published in HCL (Basso et al. 2004; Arons et al. 2020). As we had only unsorted white blood samples at our disposal, we used a special deconvolution algorithm which allowed us to select for B cell-specific genes despite the presence of contaminating other white blood cells in the samples. We could thus define a set of 17 genes specifically overexpressed in HCL. Some of the genes such as *CD19*, *CCND1* (cyclin D1), *GAS7*, *FGFR1*, *FGF2*, *RECK*, and *ITGAE* (CD103) have been published previously, based on other mRNA (RNA seq or qPCR) or protein detection methods (flow cytometry or immunohistochemistry) (Matutes et al. 1994; Bosch et al. 1995; Basso et al. 2004).

Bone marrow fibrosis, a characteristic feature of HCL, plays a crucial role in the pathogenesis of the disease and in the typical cytopenic presentation. Three genes implicated in myelofibrosis were found to be overexpressed in HLC: FGF2 (fibroblast growth factor-2; 500-fold), its receptor FGFR1 (50-fold), and *TGFB1* (tumor growth factor ß1; 16-fold).

Among the newly identified genes overexpressed in HCL, we noted specifically *RGS13* and *EBI3*. *RGS13* encodes a member of the regulator of G protein (RGS) family and is strongly expressed in germinal center (GC) B cells (Hwang et al. 2013). It was hypothesized that it might regulate the response of GC B cells to chemokines in the complex microenvironment of GCs (Shi et al. 2002). The *RGS13* chromosomal region (1q31) has been found overexpressed in HCL (Vanhentenrijk et al. 2004). *EBI3* encodes for Epstein–Barr virus (EBV)-induced gene 3, one of the two subunits, together with p35, of IL-35, an immunosuppressive cytokine of the IL-12 family (Collison et al. 2012). Larousserie et al*.* have recently shown by immunohistochemical studies in reactive lymph nodes that a subset of GC B cells located in the light zone of B cell follicles and corresponding to proliferating activated centrocytes, expressed high amounts of *EBI3* (Larousserie et al. 2019)*.*

*AICDA* coding for the activation-induced cytidine deaminase (AID) is involved in the process of somatic hypermutation which occurs during maturation of B cell receptors in the germinal center reaction. Its overexpression is also related to carcinogenesis because of off-target-induced mutations by AID, in non-immunoglobulin genes and accumulation of genetic aberration. High mRNA *AICDA* level has been previously described in cHCL, and in a lesser extent in vHCL (Hockley et al. 2010; Navarro et al. 2017). Overexpression of *EBI3*, *RGS13* as well as activation-induced cytidine deaminase (AID) encoded by *AICDA* is more suggestive of incompletely turned off GC mechanisms in post-germinal memory B cells than of a GC origin of hairy cells. Indeed, is has been shown that despite the lack of CD27 expression, HCL gene signature is closer to memory than to GC B cells (Basso et al. 2004). In favor of this hypothesis, we found that other markers commonly associated with a germinal center origin such as *BCL6*, *LMO2* and *GCSAM* (Shen et al. 2004; Natkunam et al. 2007) were also slightly downregulated in HCL compared to normal B cells.

Various hypotheses have been suggested to explain the projections of hairy cells, but the exact underlying mechanisms are still unclear: members of Rho GTPase family, such as Cdc42 and Rac1, involved in cytoskeletal rearrangement (Chaigne-Delalande et al. 2006); *GAS7* or *CD9*, both associated to microfilaments in neurons (Basso et al. 2004). More recently, the MAPK pathway inhibitor vemurafenib was shown to induce a reversion of the hairy phenotype, which was correlated with a downregulation of ß-actin and *LST1*, both proteins involved in cytoskeleton formation (Pettirossi et al. 2015). In our study, we confirmed overexpression of *GAS7* and found overexpression of *SEPTIN10* and *TTN*, both proteins interacting with cytoskeleton and actin filaments.

vHCL is considered as a provisional entity in the WHO classification (Swerdlow et al. 2017), being morphologically very similar to cHCL with the exception of the presence of a prominent nucleolus. *BRAF*^*V600E*^ is negative and *MAP2K1* mutations are identified in 42% (10/24) of cases (Waterfall et al. 2013). Immunophenotyping reveals a HCL score < 3 with a typical lack of CD25 expression. We confirmed the decreased expression of *ANXA1* (Annexin-1), and *IL2RA* (CD25) in vHCL (Swerdlow et al. 2017), as well as the overexpression of *TNFSRF13B* (Arons et al. 2020). In addition, we found that vHCL samples overexpressed the immune checkpoint regulators *CD274* (PD-L1) and *PDCD1LG2* (PD-L2) and underexpressed CD95 (FAS) receptor. These two mechanisms bypassing immune surveillance and apoptosis might reflect the more aggressive course of vHCL compared to cHCL. However, given the very small number of vHCl samples in our study (*n* = 2), these results should be interpreted with caution and be confirmed in a larger series.

The NF-κB pathway is an important regulator of B cell maturation and activation, leading to posttranslational proteolytic processing of p105 for the canonical pathway and p100 for the non-canonical pathway with induction of the active subunits p50 and p52, respectively. The canonical pathway is activated by inflammatory cytokines (e.g., IL-1) or pathogen-associated molecular patterns (PAMPs) that activate toll-like receptors, while the non-canonical pathway is mainly activated by TNF receptor family members. We found that HCL samples underexpressed the four TNF receptors *CD40*, *TNFRSF13C*, *TNFRSF17* and *TNFRSF13B* compared to normal B cells, in line with a previous report, which additionally showed underexpression of *TNFSF11*, another non-canonical pathway inducer (Basso et al. 2004). In the few samples we could analyze by Western blot, we found indications for non-canonical pathway activation with increased p100 protein processing in 5/7 samples. One explanation could be an intrinsic, receptor independent, activation of the pathway, perhaps secondary to decreased inhibitor activity. In favor of this hypothesis could be the decreased expression of the two pathway inhibitors *BIRC2* and *BIRC3* (Online Resource Table S3). A similar mechanism was proposed in CLL and MCL (Rahal et al. 2014; Diop et al. 2020). As of today, such mutations have not been described in whole exome sequencing HCL studies (Tiacci et al. 2011; Waterfall et al. 2013; Dietrich et al. 2015; Weston-Bell et al. 2016). Targeting the non-canonical pathway could constitute an interesting new approach in HCL treatment. Indeed, it has been shown in MCL and CLL, that a subgroup of cell lines resistant to classical BCR signaling and canonical pathway activation by drugs such as fludarabine and ibrutinib, respectively, exhibited non-canonical pathway activation and *BIRC3* mutations (Rahal et al. 2014; Diop et al. 2020). Activation of this pathway conferred dependence on the protein kinase NIK, a central component of the pathway. NIK could constitute a new therapeutic target for MCL treatment in cases of resistance to ibrutinib. Use of ibrutinib is an option in relapsed/refractory HCL patients (Troussard et al. 2021). In a multicenter phase 2 study, ibrutinib was evaluated in 37 patients either with cHCL (*n* = 28) or vHCL (*n* = 9 patients). The overall response rate was 24% at 32 months (Rogers et al. 2021). In patients not responding to this BTK inhibitor, the evaluation of the non-canonical NF-kB pathway could be performed and in case of activation, the use of NIK inhibitors proposed.

In conclusion, a specific mRNA signature based on the Nanostring technology was identified in HCL, which suggests a dysregulation of genes associated with germinal center and memory stages of B cell differentiation, and points towards the transformation of hairy cells occurring during the transition between the two maturation stages. We also found dysregulation of several genes associated with the non-canonical signaling and intrinsic activation of the pathway in several samples. Further studies on the non-canonical pathway are needed to determine whether targeting components of the pathway could constitute a therapeutic approach in HCL.

## Supplementary Information

Below is the link to the electronic supplementary material.

Supplementary file1 Fig S1: Fold change expression comparison between cHVL and vHCL. Scatter plot on differentially expressed genes in the 11 cHCL samples and the 2 vHCL samples compared to the 3 nB cell samples, fulfilling the following criteria: expression levels 3 20 counts, a 3 2-fold difference in expression, and a corrected p-value £0.05. The regression line the R and p value correspond to Pearson’s correlation. Fig S2: Analysis of HCL samples with the genes from the 290 Code set and annotated with GO into groups of genes ordered according to molecular function, biological process and cellular component. a: Groups of genes overexpressed in HCL samples compared to nMNC cell samples. b: Groups of genes overexpressed (upper graph) and underexpressed (lower graph) in HCL samples compared to normal B cell samples, respectively. c: Gene set enrichment analysis score and distribution of positive regulation of mitotic cycle and positive regulation of MAPK activity along the rank of transcripts differentially expressed in the HCL samples (n=13). nMNC (n=8) and nB cell samples (n=3) were used as a negative control. Figure S3: Analysis of non-canonical NF-κB pathway in HCL samples Nuclear protein extracts from patient samples were analyzed by Western blot. p100/p52 ratios were determined in six cHCL samples (pink) and one vHCL sample (blue). Two mantle cell lymphoma cell lines, REC-1 and JVM2 (green), were used as positive and negative internal controls, respectively 10. PARP served as control of nuclear protein enrichment and was obtained after stripping. Integration of volumetric signals was performed using the ImageLabTM Software and the ratio of the p100/52 volumetric signal is depicted. (PDF 1155 kb)

Supplementary file2 Table S1: Patient and samples characteristics. Origin of samples (M = Mulhouse; C = Caen; G = Geneva); Diagnosis (cHCL = classical hairy cell leukemia; vHCL = variant hairy cell leukemia; nMNC = normal mononuclear cells; nB cells = normal, purified B lymphocytes); material (PB = peripheral blood; BM = bone marrow). NA = not applicable; nd = not determined. (XLSX 11 kb)

Supplementary file3 Table S2: Gene expression values pre-normalization (GEO Access GSE66425). The table shows gene expression values (numbers of mRNA molecules) for HCL, nMNC, and nB cell samples. Values for 290 genes and 9 housekeeping genes are depicted. (XLSX 58 kb)

Supplementary file4 Table S3: 137 Genes expressed preferentially in normal B cells. Comparing expression values of the 290 genes between normal B cell and nMNC cell samples, 137 genes were found to fulfill the criteria of preferential expression in normal B cells (expression level 3 20 counts, a 3 2-fold change difference in expression compared to nMNC samples, and a corrected p-value £0.05). (XLSX 11 kb)

Supplementary file5 Table S4: List of genes expressed preferentially in HCL samples. List of genes expressed differentially in the 13 HCL samples compared to the nMNC and nB cell samples, fulfilling the following criteria: expression levels 3 20 counts, a 3 2-fold difference in expression, and a corrected p-value £0.05. a: Genes preferentially overexpressed in HCL blood samples compared to nMNC and nB cells. b: Genes preferentially underexpressed in HCL blood samples compared to nMNC and nB cells. (XLSX 14 kb)

Supplementary file6 Table S5: List of enrich ontology process involved in overexpressed and underexpressed genes in the 13 HCL samples. (Cf Xlsx file) (XLSX 17 kb)

Supplementary file7 Supplementary appendix (DOCX 14 kb)

## Data Availability

The data were submitted to GEO and can be accessed via Access Number GSE161279.

## References

[CR1] Arons E, Zhou H, Sokolsky M et al (2020) Expression of the muscle-associated gene MYF6 in hairy cell leukemia. PLoS ONE 15:e0227586. 10.1371/journal.pone.0227586 32040482 10.1371/journal.pone.0227586 PMC7010284

[CR2] Basso K, Liso A, Tiacci E et al (2004) Gene expression profiling of hairy cell leukemia reveals a phenotype related to memory B cells with altered expression of chemokine and adhesion receptors. J Exp Med 199:59–68 14707115 10.1084/jem.20031175 PMC1887727

[CR3] Beaume M, Hernandez D, Docquier M et al (2011) Orientation and expression of methicillin-resistant Staphylococcus aureus small RNAs by direct multiplexed measurements using the nCounter of NanoString technology. J Microbiol Methods 84:327–334. 10.1016/j.mimet.2010.12.025 21195730 10.1016/j.mimet.2010.12.025

[CR4] Benjamini Y, Hochberg Y (1995) Controlling the false discovery rate: a practical and powerful approach to multiple testing. J Roy Stat Soc: Ser B (methodol) 57:289–300

[CR5] Bosch F, Campo E, Jares P et al (1995) Increased expression of the PRAD-1/CCND1 gene in hairy cell leukaemia. Br J Haematol 91:1025–1030. 10.1111/j.1365-2141.1995.tb05429.x 8547115 10.1111/j.1365-2141.1995.tb05429.x

[CR6] Chaigne-Delalande B, Deuve L, Reuzeau E et al (2006) RhoGTPases and p53 are involved in the morphological appearance and interferon-α response of hairy cells. Am J Pathol 168:562–573. 10.2353/ajpath.2006.050345 16436670 10.2353/ajpath.2006.050345 PMC1606488

[CR7] Chen EY, Tan CM, Kou Y et al (2013) Enrichr: interactive and collaborative HTML5 gene list enrichment analysis tool. BMC Bioinformatics 14:128. 10.1186/1471-2105-14-128 23586463 10.1186/1471-2105-14-128 PMC3637064

[CR8] Collison LW, Delgoffe GM, Guy CS et al (2012) The composition and signaling of the IL-35 receptor are unconventional. Nat Immunol 13:290–299. 10.1038/ni.2227 22306691 10.1038/ni.2227 PMC3529151

[CR9] Cornet E, Debliquis A, Rimelen V et al (2015) Developing molecular signatures for chronic lymphocytic leukemia. PLoS ONE 10:e0128990. 10.1371/journal.pone.0128990 26046539 10.1371/journal.pone.0128990 PMC4457530

[CR10] Dietrich S, Hüllein J, Lee SC-W et al (2015) Recurrent CDKN1B (p27) mutations in hairy cell leukemia. Blood 126:1005–1008 26065650 10.1182/blood-2015-04-643361

[CR11] Diop F, Moia R, Favini C et al (2020) Biological and clinical implications of BIRC3 mutations in chronic lymphocytic leukemia. Haematologica 105:448–456. 10.3324/haematol.2019.219550 31371416 10.3324/haematol.2019.219550 PMC7012473

[CR12] Fernandez P, Solenthaler M, Spertini O et al (2012) Using digital RNA counting and flow cytometry to compare mRNA with protein expression in acute leukemias. PLoS ONE 7:e49010. 10.1371/journal.pone.0049010 23152841 10.1371/journal.pone.0049010 PMC3494663

[CR13] Geiss GK, Bumgarner RE, Birditt B et al (2008) Direct multiplexed measurement of gene expression with color-coded probe pairs. Nat Biotechnol 26:317–325. 10.1038/nbt1385 18278033 10.1038/nbt1385

[CR14] Hockley SL, Morilla A, Else M et al (2010) Higher expression levels of activation-induced cytidine deaminase distinguish hairy cell leukemia from hairy cell leukemia-variant and splenic marginal zone lymphoma. Leukemia 24:1084–1086. 10.1038/leu.2010.44 20237507 10.1038/leu.2010.44

[CR15] Hwang I-Y, Hwang K-S, Park C et al (2013) Rgs13 constrains early B cell responses and limits germinal center sizes. PLoS ONE 8:e60139. 10.1371/journal.pone.0060139 23533672 10.1371/journal.pone.0060139 PMC3606317

[CR16] Larousserie F, Kebe D, Huynh T et al (2019) Evidence for IL-35 expression in diffuse large B-cell lymphoma and impact on the patient’s prognosis. Front Oncol 9:1–12. 10.3389/fonc.2019.00563 31316915 10.3389/fonc.2019.00563 PMC6611226

[CR17] Matutes E, Morilla R, Owusu-Ankomah K et al (1994) The immunophenotype of hairy cell leukemia (HCL). Proposal for a scoring system to distinguish HCL from B-cell disorders with hairy or villous lymphocytes. Leuk Lymphoma 14:57–61 7820054

[CR18] Natkunam Y, Zhao S, Mason DY et al (2007) The oncoprotein LMO2 is expressed in normal germinal-center B cells and in human B-cell lymphomas. Blood 109:1636–1642. 10.1182/blood-2006-08-039024 17038524 10.1182/blood-2006-08-039024 PMC1794056

[CR19] Navarro A, Clot G, Martínez-Trillos A et al (2017) Improved classification of leukemic B-cell lymphoproliferative disorders using a transcriptional and genetic classifier. Haematologica 102:e360–e363. 10.3324/haematol.2016.160374 28522579 10.3324/haematol.2016.160374 PMC5685223

[CR20] Pettirossi V, Santi A, Imperi E et al (2015) BRAF inhibitors reverse the unique molecular signature and phenotype of hairy cell leukemia and exert potent antileukemic activity. Blood 125:1207–1216 25480661 10.1182/blood-2014-10-603100 PMC4366655

[CR21] Rahal R, Frick M, Romero R et al (2014) Pharmacological and genomic profiling identifies NF-κB–targeted treatment strategies for mantle cell lymphoma. Nat Med 20:87–92. 10.1038/nm.3435 24362935 10.1038/nm.3435

[CR22] Rogers KA, Andritsos LA, Wei L et al (2021) Phase 2 study of Ibrutinib in classic and variant hairy cell leukemia. Blood. 10.1182/blood.2020009688 33754642 10.1182/blood.2020009688 PMC8225920

[CR23] Shen Y, Iqbal J, Xiao L et al (2004) Distinct gene expression profiles in different B-cell compartments in human peripheral lymphoid organs. BMC Immunol 5:1–20. 10.1186/1471-2172-5-20 15369600 10.1186/1471-2172-5-20 PMC535350

[CR24] Shi G-X, Harrison K, Wilson GL et al (2002) RGS13 regulates germinal center B lymphocytes responsiveness to CXC chemokine ligand (CXCL)12 and CXCL13. J Immunol 169:2507–2515. 10.4049/jimmunol.169.5.2507 12193720 10.4049/jimmunol.169.5.2507

[CR25] Subramanian A, Kuehn H, Gould J et al (2007) GSEA-P: a desktop application for gene set enrichment analysis. Bioinformatics 23:3251–3253 17644558 10.1093/bioinformatics/btm369

[CR26] Swerdlow S, Campo E, Harris NL et al (2017) WHO classification of tumours of haematopoietic and lymphoid tissues. International agency for research on cancer, Lyon

[CR27] Tiacci E, Trifonov V, Schiavoni G et al (2011) BRAF mutations in hairy-cell leukemia. N Engl J Med 364:2305–2315 21663470 10.1056/NEJMoa1014209 PMC3689585

[CR28] Troussard X, Maitre E, Cornet E (2021) Hairy cell leukemia 2022: update on diagnosis, risk-stratification, and treatment. Am J Hematol. 10.1002/ajh.26390 34710243 10.1002/ajh.26390

[CR29] Vanhentenrijk V, De Wolf-Peeters C, Wlodarska I (2004) Comparative expressed sequence hybridization studies of hairy cell leukemia show uniform expression profile and imprint of spleen signature. Blood 104:250–255. 10.1182/blood-2004-01-0181 15016649 10.1182/blood-2004-01-0181

[CR30] Waggott D, Chu K, Yin S et al (2012) NanoStringNorm: an extensible R package for the pre-processing of NanoString mRNA and miRNA data. Bioinformatics 28:1546–1548. 10.1093/bioinformatics/bts188 22513995 10.1093/bioinformatics/bts188 PMC3356845

[CR31] Waterfall JJ, Arons E, Walker RL et al (2013) High prevalence of MAP2K1 mutations in variant and IGHV4-34–expressing hairy-cell leukemias. Nat Genet 46:8–10. 10.1038/ng.2828 24241536 10.1038/ng.2828 PMC3905739

[CR32] Weston-Bell NJ, Tapper W, Gibson J et al (2016) Exome sequencing in classic hairy cell leukaemia reveals widespread variation in acquired somatic mutations between individual tumours apart from the signature BRAF V(600)E lesion. PLoS ONE 11:e0149162. 10.1371/journal.pone.0149162 26871591 10.1371/journal.pone.0149162 PMC4752330

