# Supplementary figures and images for: Hairy cell leukemia: a specific 17-gene expression signature points to new targets for therapy
Source: J Cancer Res Clin Oncol. 2022 Apr 27;148(8):2013–22. doi: 10.1007/s00432-022-04010-4 (PMC9293816; doi:10.1007/s00432-022-04010-4)

Fig. S1

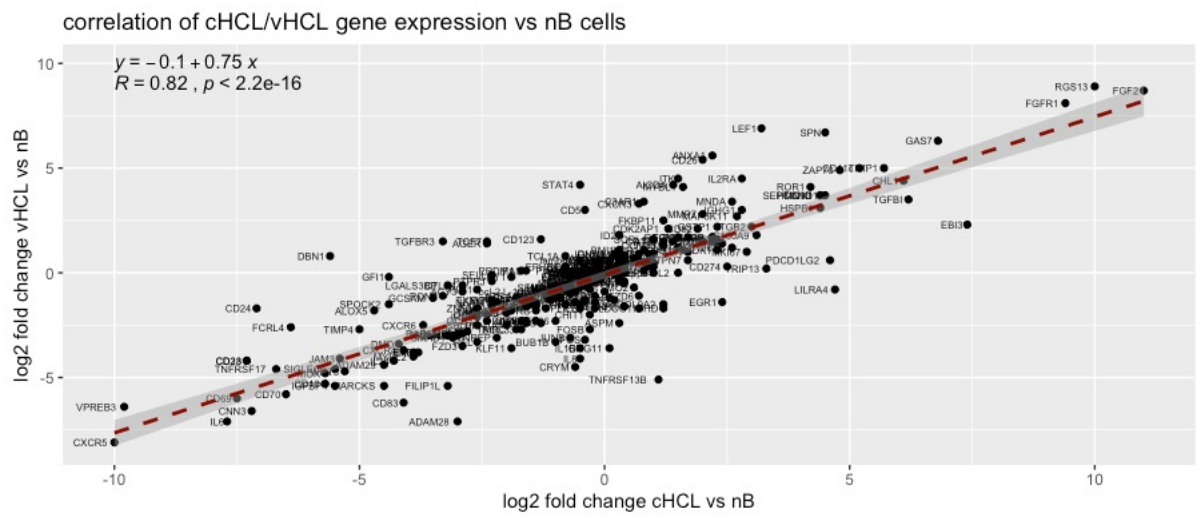

Fig. S2

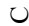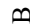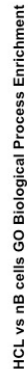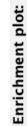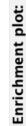

Fig. S3

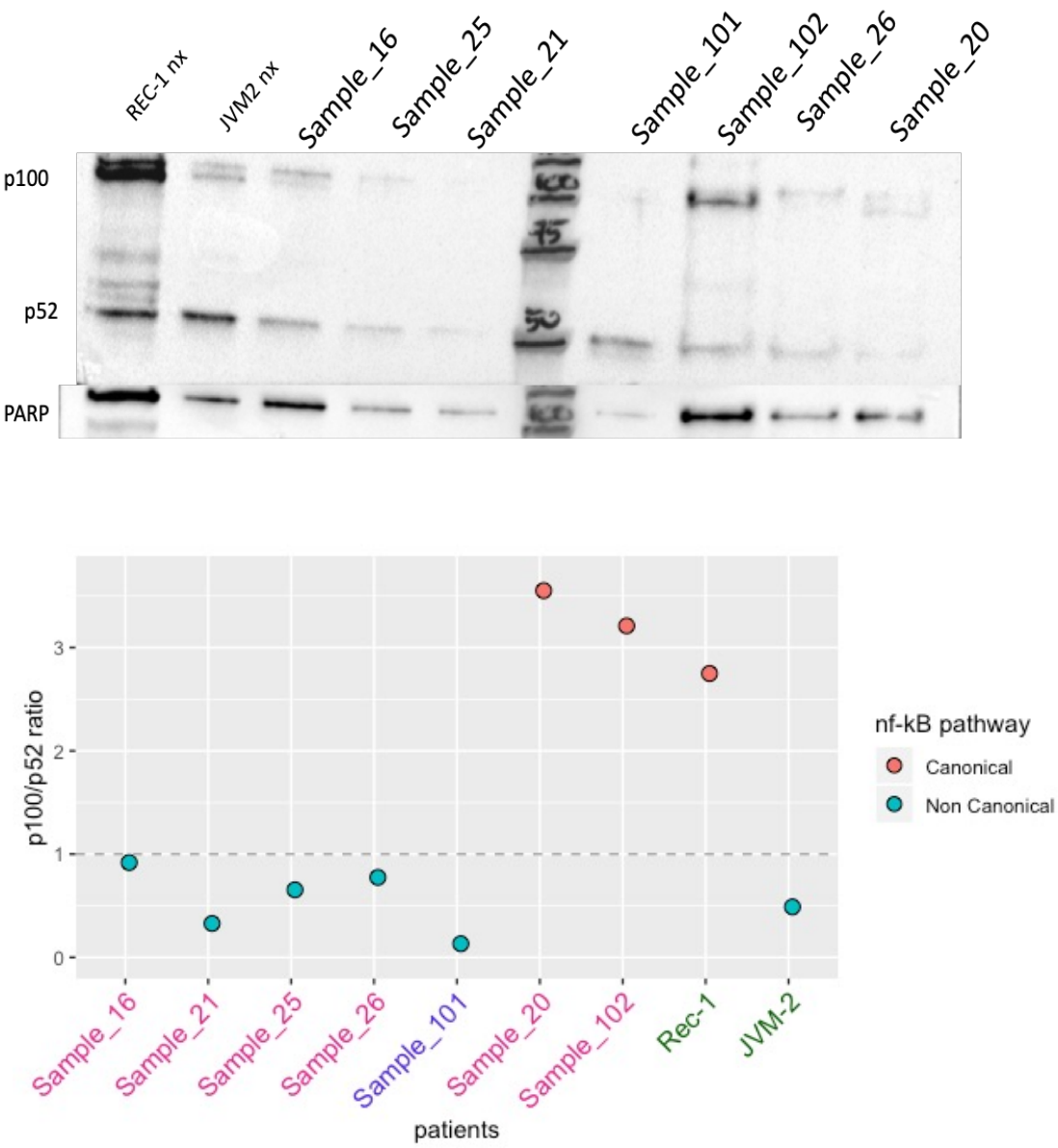

Supplement: Supplementary file 1 — Supplementary file1 Fig S1: Fold change expression comparison between cHVL and vHCL. Scatter plot on differentially expressed genes in the 11 cHCL samples and the 2 vHCL samples compared to the 3 nB cell samples, fulfilling the following criteria: expression levels 3 20 counts, a 3 2-fold difference in expression, and a corrected p-value £0.05. The regression line the R and p value correspond to Pearson’s correlation. Fig S2: Analysis of HCL samples with the genes from the 290 Code set and annotated with GO into groups of genes ordered according to molecular function, biological process and cellular component. a: Groups of genes overexpressed in HCL samples compared to nMNC cell samples. b: Groups of genes overexpressed (upper graph) and underexpressed (lower graph) in HCL samples compared to normal B cell samples, respectively. c: Gene set enrichment analysis score and distribution of positive regulation of mitotic cycle and positive regulation of MAPK activity along the rank of transcripts differentially expressed in the HCL samples (n=13). nMNC (n=8) and nB cell samples (n=3) were used as a negative control. Figure S3: Analysis of non-canonical NF-κB pathway in HCL samples Nuclear protein extracts from patient samples were analyzed by Western blot. p100/p52 ratios were determined in six cHCL samples (pink) and one vHCL sample (blue). Two mantle cell lymphoma cell lines, REC-1 and JVM2 (green), were used as positive and negative internal controls, respectively 10. PARP served as control of nuclear protein enrichment and was obtained after stripping. Integration of volumetric signals was performed using the ImageLabTM Software and the ratio of the p100/52 volumetric signal is depicted. (PDF 1155 kb) [file 432_2022_4010_MOESM1_ESM.pdf]
