# Supplementary material for: Hairy cell leukemia: a specific 17-gene expression signature points to new targets for therapy
Source: J Cancer Res Clin Oncol. 2022 Apr 27;148(8):2013–22. doi: 10.1007/s00432-022-04010-4 (PMC9293816; doi:10.1007/s00432-022-04010-4)
Supplement: Supplementary file 7 — Supplementary file7 Supplementary appendix (DOCX 14 kb) [file 432_2022_4010_MOESM7_ESM.docx]

**Supplementary appendix**

**Supplementary methods**

*Western Blot*

Cells were lysed and nuclear extracts were prepared, according to the manufacturer’s recommendations (Nuclear/Cytosol Fractionation Kit, BioVision, Milpitas, CA, USA). Mantle cell lymphoma cell lines JVM2 and REC1 were used as controls.

The protein concentration of the lysates was quantified with a Nanodrop spectrophotometer (NanoDrop One, Thermo Scientific, Madison, WI, USA). Proteins were separated on a Mini-PROTEAN® TGX^TM^ gel (BIO-RAD, Hercules, CA, USA) by SDS-PAGE and then transferred to nitrocellulose membranes using a transblot system (Trans-Blot® Turbo^TM^, BIO-RAD, Hercules, CA, USA). The membranes were incubated with the following primary antibodies: mouse anti-p52 (diluted 1:500, No. 05-361, Merck Milipore, Temecula, CA, USA), and mouse anti-PARP (diluted 1:200, No. sc-8007, Santa Cruz Biotechnology, Santa Cruz, CA, USA), followed by incubation with goat anti-mouse coupled to HRP (diluted 1:10,000, No. ab97040, Abcam, Cambridge, UK). Subsequently, the membranes were exposed to a chemiluminescence detection reagent (Clarity^TM^ Western ECL substrate, BIO-RAD, Hercules, CA, USA) and revealed on a ChemiDocTM XRS+ device (BIO-RAD, Hercules, CA, USA). Image and volumetric analyses were performed using the ImageLab^TM^ Software (version 5.2.1, BIO-RAD, Hercules, CA, USA).
